# Prox1 Directly Interacts with LSD1 and Recruits the LSD1/NuRD Complex to Epigenetically Co-Repress *CYP7A1* Transcription

**DOI:** 10.1371/journal.pone.0062192

**Published:** 2013-04-23

**Authors:** Huafang Ouyang, Yi Qin, Yanfeng Liu, Youhua Xie, Jing Liu

**Affiliations:** 1 Institute of Biochemistry and Cell Biology, Shanghai Institutes for Biological Sciences, Chinese Academy of Sciences, Shanghai, China; 2 Key Laboratory of Medical Molecular Virology (MOH & MOE) and Institutes of Biomedical Sciences, Shanghai Medical College, Fudan University, Shanghai, China; Università degli Studi di Milano, Italy

## Abstract

Cholesterol 7α-hydroxylase (CYP7A1) catalyzes the first and rate-limiting step in the classical pathway of bile acids synthesis in liver and is crucial for maintaining lipid homeostasis. Hepatocyte nuclear factor 4α (HNF4α) and α_1_-fetoprotein transcription factor (FTF) are two major transcription factors driving *CYP7A1* promoter activity in hepatocytes. Previous researches have shown that Prospero-related homeobox (Prox1) directly interacts with both HNF4α and FTF and potently co-represses *CYP7A1* transcription and bile acid synthesis through unidentified mechanisms. In this work, mechanisms involved in Prox1-mediated co-repression were explored by identifying Prox1-associated proteins using immunoprecipitation followed by mass spectrometry (IP-MS) methodology. Multiple components of the epigenetically repressive lysine-specific demethylase 1 (LSD1)/nucleosome remodeling and histone deacetylase (NuRD) complex, most notably LSD1 and histone deacetylase 2 (HDAC2), were found to be associated with Prox1 and GST pulldown assay demonstrated that Prox1 directly interacts with LSD1. Sequential chromatin immunoprecipitation (ChIP) assays showed that Prox1 co-localizes with HNF4α, LSD1 and HDAC2 on *CYP7A1* promoter in HepG2 cells. Furthermore, by using ChIP assay on HepG2 cells with endogenous Prox1 knocked down by RNA interference, Prox1 was shown to recruit LSD1 and HDAC2 onto *CYP7A1* promoter and cause increased H3K4 demethylation. Finally, bile acids treatment of HepG2 cells, which significantly repressed *CYP7A1* transcription, resulted in increased Prox1 and LSD1/NuRD complex occupancy on *CYP7A1* promoter with a concurrent increase in H3K4 demethylation and H3/H4 deacetylation. These results showed that Prox1 interacts with LSD1 to recruit the repressive LSD1/NuRD complex to *CYP7A1* promoter and co-represses transcription through epigenetic mechanisms. In addition, such Prox1-mediated epigenetic repression is involved in the physiologically essential negative feedback inhibition of *CYP7A1* transcription by bile acids.

## Introduction

Bile acids (BA) are synthesized in the liver and function as physiological detergents that facilitate intestinal absorption and transport of lipids, nutrients and vitamins, as well as disposal of toxic metabolites and xenobiotics [1]–[3]. Bile acids have also been recognized as important signaling molecules and inflammatory agents that regulate lipid, glucose, and energy metabolism [1]. Cholesterol 7α-hydroxylase (CYP7A1) is the enzyme that catalyzes the first and rate-limiting step in the classical pathway of bile acids synthesis from cholesterol, which accounts for 90% of total BA production in human liver [4]. As such, CYP7A1 plays a pivotal role in maintaining lipid homeostasis *in vivo* by responding to various physiological conditions and signals with varying expression levels [1]–[4].

*CYP7A1* mRNA has been shown to be short-lived [5], [6] and regulation of CYP7A1 expression occurs mainly at transcription level [1], [4]. Two bile acid response elements BARE-I and BARE-II have been identified upstream of *CYP7A1* promoter: BARE-I of rat and mouse, but not human or other non-rodent species, contains binding site for liver X receptor α (LXRα, NR1H3)/retinoic acid receptor (RXR) heterodimer, which is capable of activating *CYP7A1* expression in response to oxysterol [7], [8]; BARE-II is highly conserved among species and contains overlapping binding sites for transcription activators α_1_-fetoprotein transcription factor (FTF, NR5A2) [9] and hepatocyte nuclear factor-4α (HNF4α, NR2A1) [10]. Transcriptional activation by HNF4α requires co-activators including peroxisome proliferator-activated receptor γ co-activator 1α (PGC-1α) [11], [12], steroid receptor coactivator-1 (SRC-1) [11] and chicken ovalbumin upstream promoter transcription factor II (COUP-TFII) [13], while activation of *CYP7A1* promoter by both FTF and HNF4α is subjected to negative regulation by co-repressors such as atypical nuclear small heterodimer partner (SHP, NR0B2) [14], [15].

Most *CYP7A1* transcription regulation mechanisms in hepatocytes studied so far directly or indirectly target FTF, HNF4α and co-activators/co-repressors acting through them [1], [4]. Inhibition of hepatocyte CYP7A1 expression by bile acids returning from small intestine to liver via enterohepatic bile circulation constitutes a negative feedback loop essential for lipid homeostatis *in vivo* [1], [2], [4]. Mechanistic studies identified farnesoid X receptor (FXR, NR1H4) as the major hepatocyte bile acid receptor involved in bile acid-mediated *CYP7A1* repression [16]. Engagement of FXR with ligands could induce SHP transcription and elevated SHP expression in turn co-represses both FTF and HNF4α to reduce *CYP7A1* transcription [15], [17].

Prospero-related homeobox (Prox1) is the vertebrate homolog of *Drosophila melanogaster* Prospero transcription factor and mainly expressed in lens, heart, liver, kidney, spleen, skeletal muscle, pancreas and the central nervous system [18]. Previous studies have demonstrated that Prox1 is essential for the development of lens [19], lymphatic system [20] and liver [21], and might be involved in carcinogenesis in certain tissue types [22]. In humans, Prox1 has also been shown to participate in host-pathogen interactions [23], [24]. Expression of multiple genes in various tissues is apparently affected by Prox1, but the underlying molecular mechanisms have not been studied in detail in most cases. Despite the presence of a C-terminal Prospero/homeobox domain, which mediates DNA-binding in Prospero and other related proteins [25], Prox1 has only been shown to bind directly to promoter DNA sequences in rare cases [26].

Work conducted in our laboratory identified Prox1 as physically interacting with FTF and co-repressing the latter’s activation of *CYP7A1* in cultured hepatocytes [27]. Similar mechanisms were also demonstrated for the other key activator of *CYP7A1,* HNF4α, whereby Prox1 interacts and co-represses transcriptional activation of *CYP7A1* by HNF4α [28]. Although Prox1 does not bind *CYP7A1* promoter directly [27], [28], co-repression of the promoter activity through both FTF and HNF4α makes Prox1 an important co-regulator of *CYP7A1* transcription and bile acid synthesis. *In vitro*, knockdown of Prox1 expression using RNA interference indeed resulted in elevated *CYP7A1* mRNA level and bile acid synthesis activity in cultured hepatocytes [28]. Mechanisms underlying Prox1-mediated co-repression of *CYP7A1* transcription are not yet fully understood. For co-repression of HNF-4α, there have been results indicating that Prox1 might interfere with the recruitment of PGC-1α co-activator by HNF4α [28]. Involvement of epigenetic mechanisms has also been suspected, due to the apparent interaction and co-localization between Prox1 and histone deacetylase 3 (HDAC3) [29].

In this work, we attempted to delineate some of the mechanisms involved in Prox1-mediated co-repression of *CYP7A1* and started by identifying Prox1-associated proteins using immunoprecipitation followed by mass spectrometry (IP-MS) method. Multiple components of lysine-specific demethylase 1 (LSD1)/nucleosome remodeling and histone deacetylase (NuRD) complex, most notably LSD1 and histone deacetylase 2 (HDAC2), were found to be associated with Prox1 in hepatocytes. Co-immunoprecipitation (co-IP) and GST pulldown assays indicated that Prox1 directly interacts with LSD1. In HepG2 cells as well as mouse liver cells, chromatin immunoprecipitation (ChIP) assays revealed the occupancies of Prox1, HNF4α, LSD1 and HDAC2 on *CYP7A1* promoter. Moreover, sequential ChIP assays showed that Prox1 co-localizes with HNF4α, LSD1 and HDAC2 on *CYP7A1* promoter in HepG2 cells. We then provide evidences showing that Prox1 recruits LSD1 and HDAC2 onto *CYP7A1* promoter and corresponding repressive changes in histone modification status were rendered. We also show that Prox1-mediated LSD1/NuRD complex recruitment is involved in BA-induced *CYP7A1* repression. Results presented here reveal novel epigenetic mechanisms involved in Prox1-mediated co-repression of *CYP7A1* transcription.

## Materials and Methods

### Ethics Statement

Handling of animals conformed to the guidelines approved by the Animal Ethics Committee of Shanghai Medical College, Fudan University and the protocol was approved by the Committee (Permit Number: 20101201-001).

### Plasmid Constructs

FLAG-tagged full-length Prox1 was cloned in pcDNA3 (Invitrogen) to create pFLAG-Prox1. Lentiviral vectors pLKO.1 TRC (Addgene plasmid 10879) [30] and pWPI.1 (Addgene plasmid 12254) were used for producing recombinant lentiviruses to achieve RNA interference (RNAi) and overexpression respectively. For RNAi of human *PROX1*, si258 (5′-TTTCCAGGAGCAACCATAATT-3′) and si1646 (5′-GGCTCTCCTTGTCGCTCATAA-3′), were inserted as hairpin precursors into pLKO.1 TRC. A scrambled RNAi precursor (siSCR) possessing similar GC-content to si258 and si1646 but no sequence identity with *PROX1* was used as negative control. For overexpression of Prox1, full-length Prox1 cDNA (FLAG-Prox1) was cloned into pWPI.1. Synonymous mutations were introduced at si258 (5′-TTTCCAGGAGCTACTATCATC-3′, mutations underlined) and si1646 target sequences (5′-GGCTCTCATTATCACTCATAA-3′, mutations underlined) in Prox1 coding sequences to create the RNAi resistant pWPI.1-Prox1m.

### Cell Lines, Lentiviruses and Animals

Human hepatoblastoma cell line HepG2 and embryo kidney cell line HEK293T were purchased from Cell Bank of Shanghai Institutes of Biological Sciences (SIBS), Chinese Academy of Sciences (CAS). Cells were maintained in Dulbecco’s modified Eagle medium (DMEM) (Invitrogen) supplemented with 100 U/ml penicillin G/streptomycin sulfate and 10% (v/v) fetal bovine serum (Invitrogen), and cultured at 37°C with 5% CO_2_. Transfections were performed using plasmid DNA and polyethylenimine (Sigma) at 1∶1 ratio. For chenodeoxycholic acid (CDCA) treatment, HepG2 cells were changed into serum-free DMEM containing 25 µmol/L CDCA (Sigma) and cultured for 16 hours.

Helper plasmids pSPAX2 (Addgene plasmid 12260) and pMD2.G (Addgene plasmid 12259) were co-transfected with pLKO.1- or pWPI.1-based plasmids into HEK293T cells to package recombinant lentiviruses. Supernatants from co-transfections were used directly for infection of cultured cells.

BALB/c mice were purchased from Shanghai Laboratory Animal Center (SLAC) of SIBS, CAS and sacrificed by cervical dislocation. Liver was surgically removed and approximately 1 g liver tissue was subjected to homogenization using a mechanical homogenizer before ChIP analysis.

### Immunoprecipitation and Mass Spectrometry

HEK293T cells transfected with pFLAG-Prox1 expression plasmid were lysed in RIPA buffer (20 mM Tris pH 8.0, 137 mM NaCl, 1% NP40, 10% Glycerol, 2 mM EDTA) and the lysate was cleared by centrifugation at 12000 *g* before being applied to M2 anti-FLAG monoclonal antibody agarose beads (Sigma) pre-equilibrated in RIPA buffer. The beads were washed using RIPA buffer and bound proteins eluted using 3xFLAG peptide (Sigma). Both eluants and post-elution beads were boiled in loading buffer, resolved on denaturing SDS-PAGE and silver stained. Lysates from HEK293T cells transfected with empty vector were used as control and processed in parallel. Bands specific to pFLAG-Prox1 transfected HEK293T were excised and subjected to MS analysis on ABI 4700 MALDI TOF/TOF.

### Co-immnunoprecipitation and GST Pulldown

Co-IP was performed by lysing HEK293T cells transfected with pFLAG-PROX1 and HepG2 cells in RIPA buffer and lysates were cleared by centrifugation at 12000 *g* for 10 min. FLAG-tagged Prox1 was precipitated as described above for IP-MS, whereas endogenous Prox1 in HepG2 was precipitated using anti-Prox1 antibody (Upstate) bound to protein A/G agarose beads (GE Healthcare). Beads were washed with RIPA buffer, boiled in loading buffer and resolved on denaturing SDS-PAGE. Prox1 associated proteins were detected using Western blot and antibodies against Mi2, MTA2, RbAp46, MBD3, HDAC2 (Santa Cruz) and HNF4α (Abcam).

For GST pulldown assay, GST and GST-fused Prox1 fragments were expressed in *Escherichia coli* BL21(DE3) strain and purified using glutathione-Sepharose 4B beads (GE Healthcare Bio-sciences) according to manufacturer's protocol. LSD1 and HDAC2 proteins were *in vitro* translated from corresponding plasmids using TNT-coupled transcriptional translation system (Promega). Glutathione-Sepharose beads with bound GST or GST fusion proteins were incubated with 50 µl *in vitro* translation products in 450 µl binding buffer [20 mM Tris pH 8.0, 137 mM NaCl, 1% NP40, 10% Glycerol, 2 mM EDTA, 1 mM PMSF] at 4°C for 4 h. Beads were washed with wash buffer [20 mM Tris pH 8.0, 250 mM NaCl, 1% NP40, 10% Glycerol, 2 mM EDTA, 1 mM PMSF], boiled in SDS-PAGE loading buffer and analyzed in Western blot.

### RNA Isolation and Quantitative Real-time PCR (qrtPCR)

Total RNA was isolated from HepG2 cells using Trizol (Invitrogen) and approximately 2 µg RNA was reverse-transcribed using RETROscript (Ambion) according to manufacturers' protocols. Aliquots of cDNA were subjected to real-time PCR using Taqman probes for *CYP7A1* (Hs00167982), *PROX1* (Hs00896294) and *UBC* (Hs00824723) (Applied Biosystems) and Taqman Universal PCR Master Mix (Roche) following manufacturer’s instructions. All PCR reactions were done in triplicates using conditions as follows: 50°C/2 min, 95°C/10 min, 40 cycles of 95°C/15 s and 60°C/1 min on MXP3000 cycler (Stratagene) and repeated at least 3 times. Relative mRNA levels were calculated using the –ΔΔCt method using *UBC* as control and expressed as 2∧(–ΔΔCt).

### Bile Acid Measurement

HepG2 cells were suspended in 2∶1 chloroform/methyl alcohol and vigorously vortexed. Hydrophilic bile acids were extracted by adding 1/3 volume H_2_O followed by vortexing and centrifugation. Bile acids in the aqueous phase were measured using a bile acid colorimetric assay (Diasys Diagnostic Technology) following the manufacturer’s instructions. To control for input cell mass variations, total phospholipids in the organic phase were measured using a phospholipid colorimetric assay (Kinghawk Pharmaceutical) following the manufacturer’s instructions.

### Chromatin Immunoprecipitation (ChIP) Assay

ChIP assays were performed following a published protocol [31]. Antibodies against Prox1 (Upstate Biotechnology, 07-537), LSD1 (Abcam, ab17721), HDAC2 (Abcam, ab7029), HNF4α (Abcam, ab41898), dimethyl-Histone H3 (Millipore, 07-030), acetyl-Histone H3 (Millipore, 06-599), acetyl-Histone H4 (Millipore, 06-598), SRC1 (Santa Cruz, sc-6098), p300 (Santa Cruz, sc-585) and CBP (Santa Cruz, sc-369) were used to immuno-precipitate sonicated chromatin prepared from cultured cells or mouse liver cells. Five percent (5%) of post-sonication sample was saved as input control and normal (pre-immnue) IgG was used for specificity control. DNA extracted from precipitated chromatin were quantitated using qrtPCR in triplicates using primers for human (forward, 5'-AGCTGTTGTCCCCAGGTCCGA-3'; reverse, 5'-TCCACAGGTATCAGAAGTGGTTCCA-3') or mouse (forward, 5'-ACCTTCGGCTTATCGACTATTGC-3'; reverse, 5'-TATCTGGCCTTGAACTAAGTCCATCT-3') *CYP7A1* promoter as previously described [28], [32]. Primers annealing to a downstream mRNA-encoding region (forward, 5′-GAACCACCTCTAGAGAATG-3′, reverse, 5′-GAATCTCCACATAAGGATAAC-3′) were used in parallel as negative occupancy control also as previously described [33]. DNA extracted from saved input sample were quantitated in parallel (Ct[Input]) and adjusted to 100% using the equation: Adjusted Ct[Input] = Ct[Input] − 4.322. (log_2_(5%) = −4.322). Results for IP by normal IgG or specific antibody (Ct[IP]) were then used to calculate relative non-specific background and specific occupancy using the equation: 2∧(Adjusted Ct[Input]−Ct[IP]) * 100% [32].

For sequential ChIP (re-ChIP) assay, after the first round precipitation using anti-Prox1 antibodies, beads were incubated with equal volume of 10 mM DTT for 30 minutes at 37°C, centrifuged and supernatants containing precipitated chromatin fragments were transferred into new tubes. The eluted samples were diluted 50 times with IP buffer and 5% of the sample was saved as input control. Second round ChIP was then performed according to standard protocol as described above.

### Statistical Analysis

ChIP and qrtPCR results from three independent experiments were analyzed using student’s *t*-test and *P* values smaller than 0.05 were considered significant.

## Results

### Prox1 Represses *CYP7A1* Transcription and Bile Acid Synthesis in HepG2 Cells

**Figure 1:**
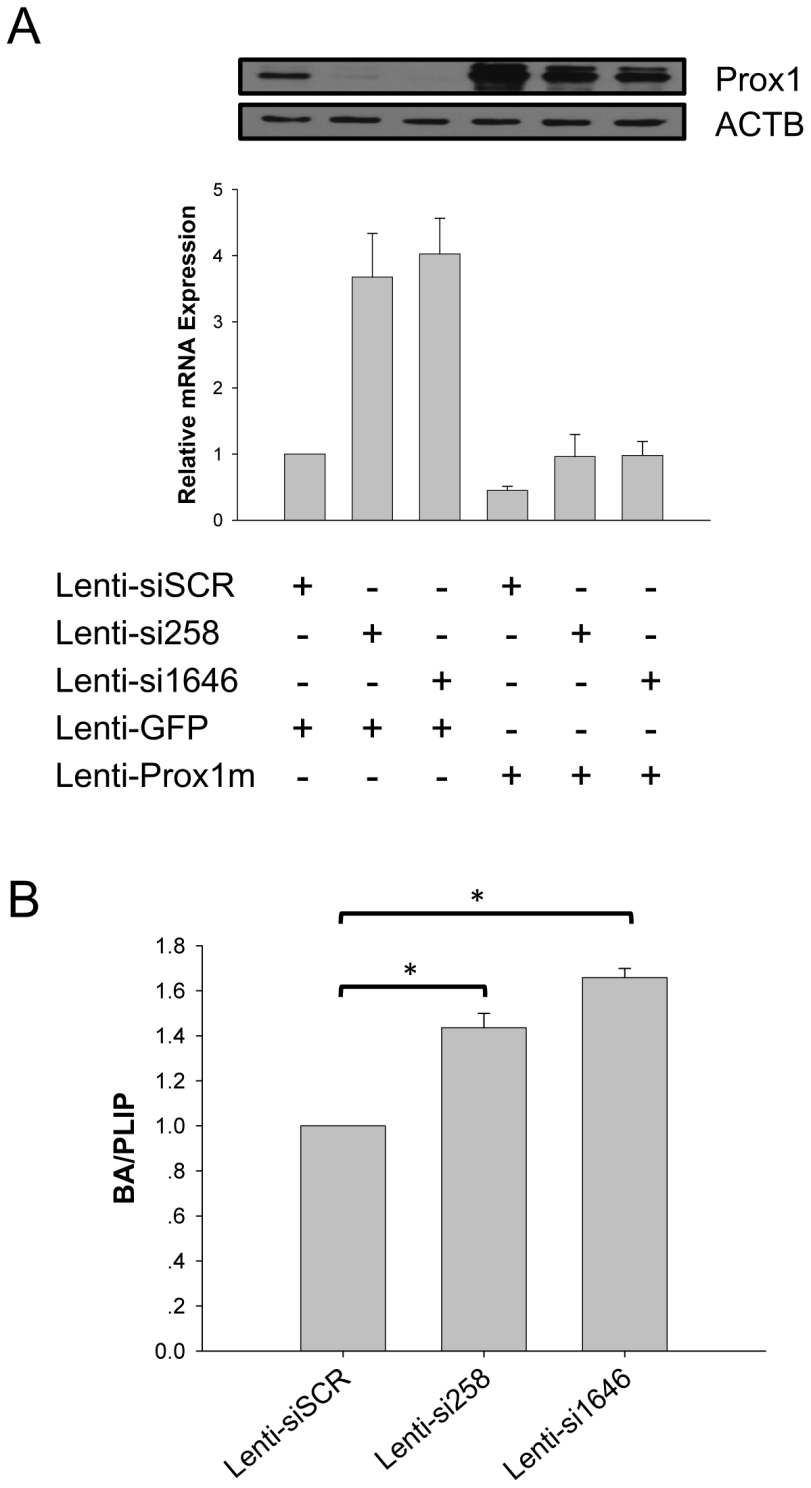
Prox1 represses *CYP7A1* transcription and bile acid synthesis in HepG2 cells. (A) Prox1 represses transcription of *CYP7A1* mRNA. HepG2 cells were co-infected with recombinant lentiviruses expressing Prox1-targeting siRNA precursors si258 or si1646, or scrambled control siSCR, and recombinant lentiviruses expressing control GFP or siRNA-insensitive Prox1 mutant Prox1m as indicated. Total RNA was extracted 36 hrs post-infection and levels of *CYP7A1* mRNA measured using quantitative real-time PCR as described in Materials and Methods. Means and SD from three independent experiments are presented. Prox1 expression levels were analyzed in Western blot using beta-actin as loading control (top). (B) Prox1 represses BA synthesis. HepG2 cells were infected with recombinant lentiviruses expressing Prox1-targeting siRNA precursors si258 or si1646, or scrambled control siSCR. Total intracellular BA and phospholipids were extracted and measured as described in Materials and Methods. Relative bile acid levels are expressed as BA/phospholipids and presented, taking result from lenti-siSCR-infected cells as 1. Means and SD from three independent experiments are presented. Statistically significant changes (*P<*0.05 in student’s *t* test) were indicated (*).

In order to explore mechanisms underlying Prox1-mediated co-repression of *CYP7A1* transcription, we first reconfirmed such repression in cultured HepG2 cells using lentivirus-mediated knockdown and rescue of Prox1 expression. Infection with lentiviruses expressing Prox1-targeting siRNA precursors si258 (lenti-si258) or si1646 (lenti-si1646) nearly obliterated endogenous Prox1 expression (Fig. 1A, top, lanes 1–3), and caused a marked increase in *CYP7A1* mRNA level (Fig. 1A, bottom, bars 1–3). Co-infection with lentivirus expressing RNAi-resistant Prox1 mutant (lenti-Prox1m) reinstated Prox1 expression (Fig. 1A, top, lanes 5–6), and intracellular *CYP7A1* mRNA level also returned to a level comparable to HepG2 cells infected with control lentiviruses (Fig. 1A, bottom, bars 5–6). On the other hand, infection by lenti-Prox1m resulted in overexpression of Prox1 (Fig. 1A, top, lane 4) and decrease in *CYP7A1* mRNA level (Fig. 1A, bottom, bar 4). When BA synthesis was analyzed, lenti-si258 or lenti-si1646 infected HepG2 cells displayed elevated BA production activity compared to cells infected with control virus (Fig. 1B), in accordance with increased *CYP7A1* transcription (Fig. 1A, bottom, bars 2–3). These results were in good agreement with previous reports and reconfirmed the repressive effects of Prox1 on *CYP7A1* transcription and, consequently, downstream bile acid synthesis.

### Prox1 is Associated with LSD1/NuRD Complex and Directly Interacts with LSD1

**Figure 2:**
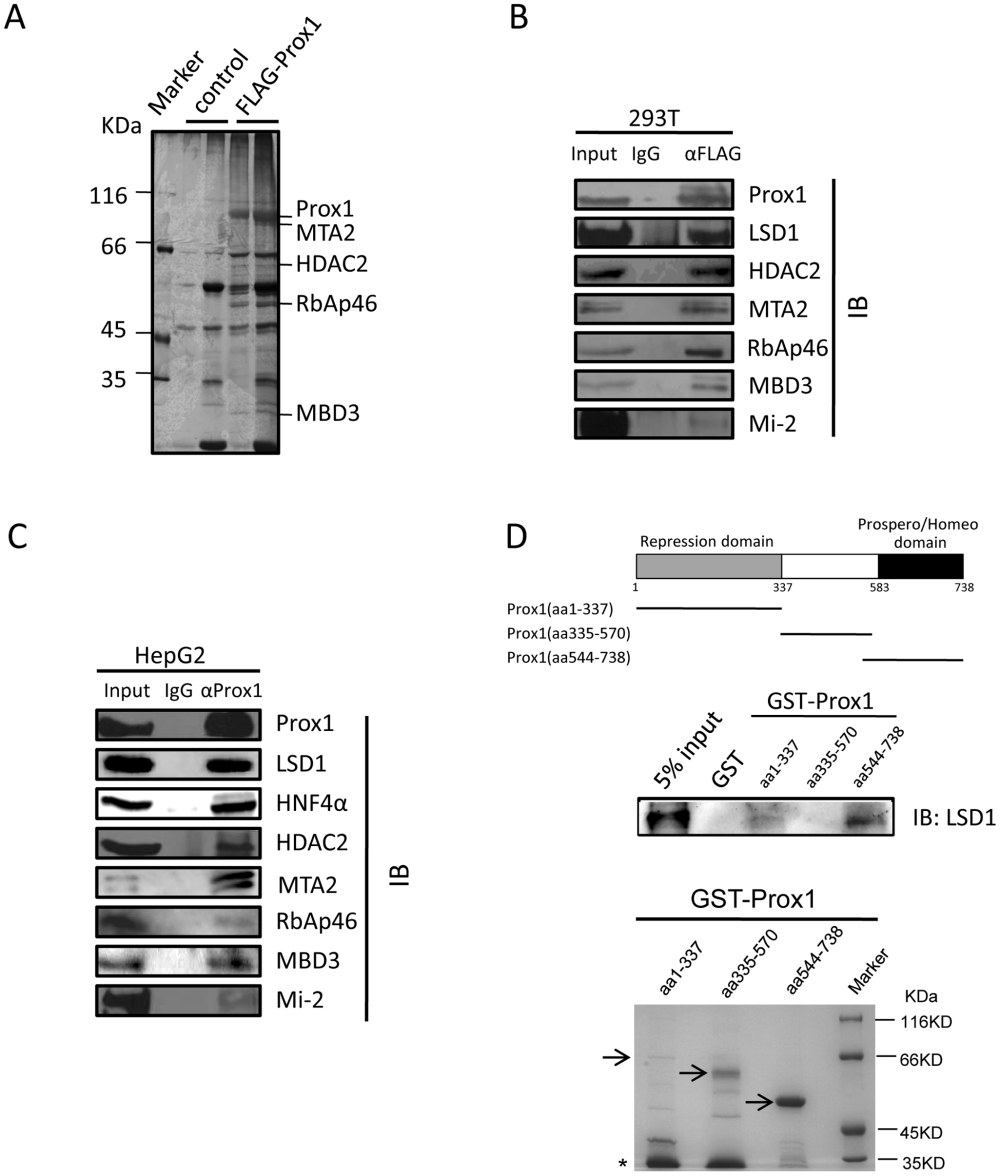
Prox1 is associated with LSD1/NuRD complex and directly interacts with LSD1. (A) Identification of Prox1-associated proteins using immunoprecipitation and mass spectrometry (IP-MS). HEK293T cells were transfected with plasmid expressing FLAG-tagged Prox1 and Prox1-associated proteins were immunoprecipitated using anti-FLAG monoclonal antibodies. Cells transfected with empty vector were processed in parallel as negative control. Precipitated proteins were resolved on denaturing SDS-PAGE and silver-stained. Bands exclusively found in FLAG-Prox1 samples were excised and identified using MS. Positions of bands corresponding to Prox1 and multiple LSD1/NuRD complex components are indicated. (B) Association of exogenous Prox1 with LSD1/NuRD complex in HEK293T cells. HEK293T cells transfected with plasmid expressing FLAG-tagged Prox1 or empty vector were subjected to co-immunoprecipitation assay using anti-FLAG monoclonal antibodies. Co-immunoprecipitated proteins were detected in Western blot using antibodies to LSD1/NuRD complex components as indicated. One tenth of cell lysate before co-immunoprecipitation was used as input control. (C) Association of endogenous Prox1 with LSD1/NuRD complex in HepG2 cells. HepG2 cells were subjected to co-immunoprecipitation assay using anti-Prox1 antibodies. Co-immunoprecipitated HNF4α and LSD1/NuRD complex components were detected in Western blot using corresponding antibodies as indicated. (D) Prox1 directly interacts with *in vitro* translated LSD1 in GST pulldown assay. Schematic representation of Prox1 domain organization is depicted (top). GST-fused repression (aa 1–337), central (aa 335–570) and Prospero/homeo (aa 544–738) domains of Prox1 were expressed in *E. coli* BL21(DE3) and purified using Glutathione-Sepharose beads. Beads with bound GST-Prox1 proteins were then incubated *in vitro* translated LSD1 and LSD1 pulled down was detected using Western blot. GST was used as negative control.

Previous work has shown that Prox1 represses *CYP7A1* transcription by functioning as a co-repressor of transcriptional activators FTF and HNF4α [27], [28]. To probe for molecules involved in this process, we started by identifying Prox1-associated proteins using IP-MS methodology. FLAG-tagged Prox1 was over-expressed in HEK293T cells and immunoprecipitated using anti-FLAG monoclonal antibody. Co-immunoprecipitated proteins were visualized after electrophoresis using silver staining and identified through mass spectrometry analysis (Fig. 2A). Interestingly, multiple components of the repressive LSD1/NuRD complex [34], including HDAC2, RbAp46, MBD3 and MTA2, were identified by IP-MS (Fig. 2A and Supplementary Table S1). Association of a majority of known components of LSD1/NuRD complex, including LSD1, HDAC2, Mi-2, RbAp46, MBD3 and MTA2 with FLAG-tagged Prox1 in HEK293T was then confirmed using conventional co-IP methods (Fig. 2B).

As HEK293T lacks endogenous Prox1 expression [35], we went on to test whether endogenously expressed Prox1 in HepG2 is also associated with LSD1/NuRD components. Co-IP of HepG2 lysates using anti-Prox1 antibody demonstrated that endogenous Prox1 in HepG2 is indeed associated with LSD1 and HDAC2, as well as Mi-2, RbAp46, MBD3 and MTA2 (Fig. 2C), corroborating results obtained with exogenous Prox1 in HEK293T (Fig. 2A and 2B). In addition, such associations were not affected by DNase/RNase treatment (Supplementary Fig. S2).

GST pulldown assay was then used to determine whether there exist any direct interactions between Prox1 and the associated LSD1/NuRD components. As full-length Prox1 is difficult to express and purify in *E. coli* [23], [28], fragments of Prox1 was expressed as GST-fusion proteins and used as bait to pull down *in vitro* translated LSD1, MTA2 and HDAC2, respectively. LSD1 could be successfully pulled down by both N-terminal (aa 1–337) and C-terminal (aa 544–738) segments of Prox1, which encompass the repression domain and Prospero/homeobox domain respectively, but not by the central (aa 335–570) segment (Fig. 2D). No interactions between Prox1 and MTA2 or HDAC2 could be observed in GST pulldown (data not shown). Direct interaction between LSD1 and Prox1 suggests that Prox1 is associated with LSD1/NuRD complex through directly binding LSD1, although it can’t be ruled out that Prox1 might also interact with other NuRD complex components that were not tested.

### Prox1 and LSD1/NuRD Complex Co-localize on Human and Mouse CYP7A1 Promoter

**Figure 3:**
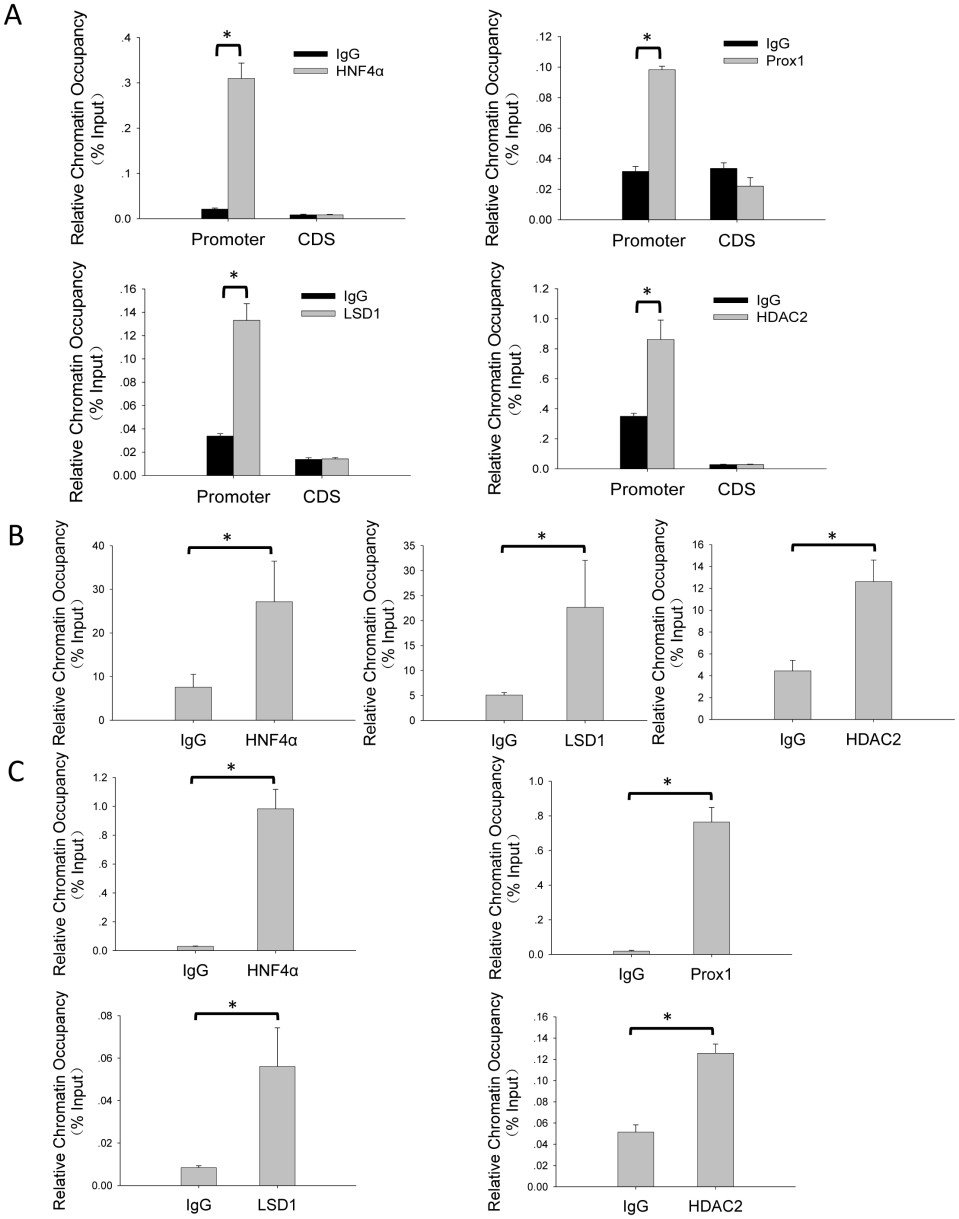
Prox1 co-localizes with LSD1/NuRD complex components on *CYP7A1* promoter. (A) Occupancy of HNF4α, Prox1, and LSD1/NuRD components on *CYP7A1* promoter in HepG2 cells. Chromatin immunoprecipitation (ChIP) was performed on chromatin fragments prepared from HepG2 cells using specific antibodies as indicated and corresponding normal IgG as non-specific control. (B) Prox1 co-localizes with LSD1 and HDAC2 on *CYP7A1* promoter in HepG2 cells. Sequential ChIP was performed on chromatin fragments prepared from HepG2 cells using anti-Prox1 antibodies for first round ChIP (A) and antibodies to HNF4α LSD1 and HDAC2 for second round ChIP, respectively. (C) Occupancy of Prox1, LSD1 and HDAC2 on *CYP7A1* promoter in mouse liver cells. Chromatin fragments prepared from mouse liver cells were subjected to ChIP using antibodies to Prox1, HNF4α LSD1 and HDAC2 as indicated. In panels A-C, corresponding normal mouse or rabbit IgG was used as non-specific background control for each antibody used. Precipitated *CYP7A1* promoter segments were detected using quantitative real-time PCR and relative chromatin occupancy was calculated as %input as described in Materials and Methods. In panel A, a control region in downstream *CYP7A1* mRNA coding sequences (CDS) was also quantitated using real-time PCR in parallel as further demonstration of assay specificity. Means and SD from three independent experiments are presented. Statistically significant enrichment by specific antibodies (*P<*0.05 in student’s *t* test) were indicated (*).

Since Prox1 directly binds LSD1 and can be associated with LSD1/NuRD complex, we wondered whether such interactions would enable Prox1 to recruit LSD1/NuRD complex onto the promoter of *CYP7A1*. To explore such a possibility, we first demonstrated in HepG2 cells, using ChIP assay, occupancy of Prox1 and HNF4α on human *CYP7A1* promoter segment (−432 to −41) harboring the overlapping FTF/HNF4α binding site [28], but not on a downstream region within mRNA coding sequences [33] used as negative control (Fig. 3A, top panels). These results are in agreement with previously published results [28], [33]. ChIP assay also identified LSD1 and HDAC2 as occupant on the same segment of *CYP7A1* promoter but not on the downstream control region (Fig. 3A). Similarly, ChIP performed on chromatin prepared from mouse liver cells demonstrated occupancy of HNF4α Prox1, LSD1 and HDAC2 on the corresponding segment (−219 to −163) of mouse *CYP7A1* promoter [36] (Fig. 3C).

Sequential ChIP-reChIP assay was then used to test whether there is any co-occupancy between Prox1 and LSD1/NuRD complex on *CYP7A1* promoter in HepG2 cells. Chromatin fragments immunoprecipitated by anti-Prox1 antibody (Fig. 3A, top right) were subjected to second round immunoprecipitation using antibodies to HNF4α, LSD1 or HDAC2, respectively, all of which specifically enriched *CYP7A1* promoter DNA compared to respective non-specific IgG controls(Fig. 3B). This result indicated that Prox1 could co-localize with HNF4α, as well as LSD1/NuRD components LSD1 and HDAC2, on human *CYP7A1* promoter. Since HNF4αbinds *CYP7A1* promoter directly whereas Prox1 does not [28], co-occupancy of these two factors confirmed that co-repressor Prox1 could be recruited by HNF4αto *CYP7A1* promoter [28]. On the other hand, co-occupancy of Prox1 with LSD1 and HDAC2 suggested that Prox1 might in turn recruit LSD1/NuRD complex components onto *CYP7A1* promoter.

### Prox1 Recruits LSD1/NuRD Complex to *CYP7A1* Promoter to Exert Epigenetic Repression of *CYP7A1* Transcription

**Figure 4:**
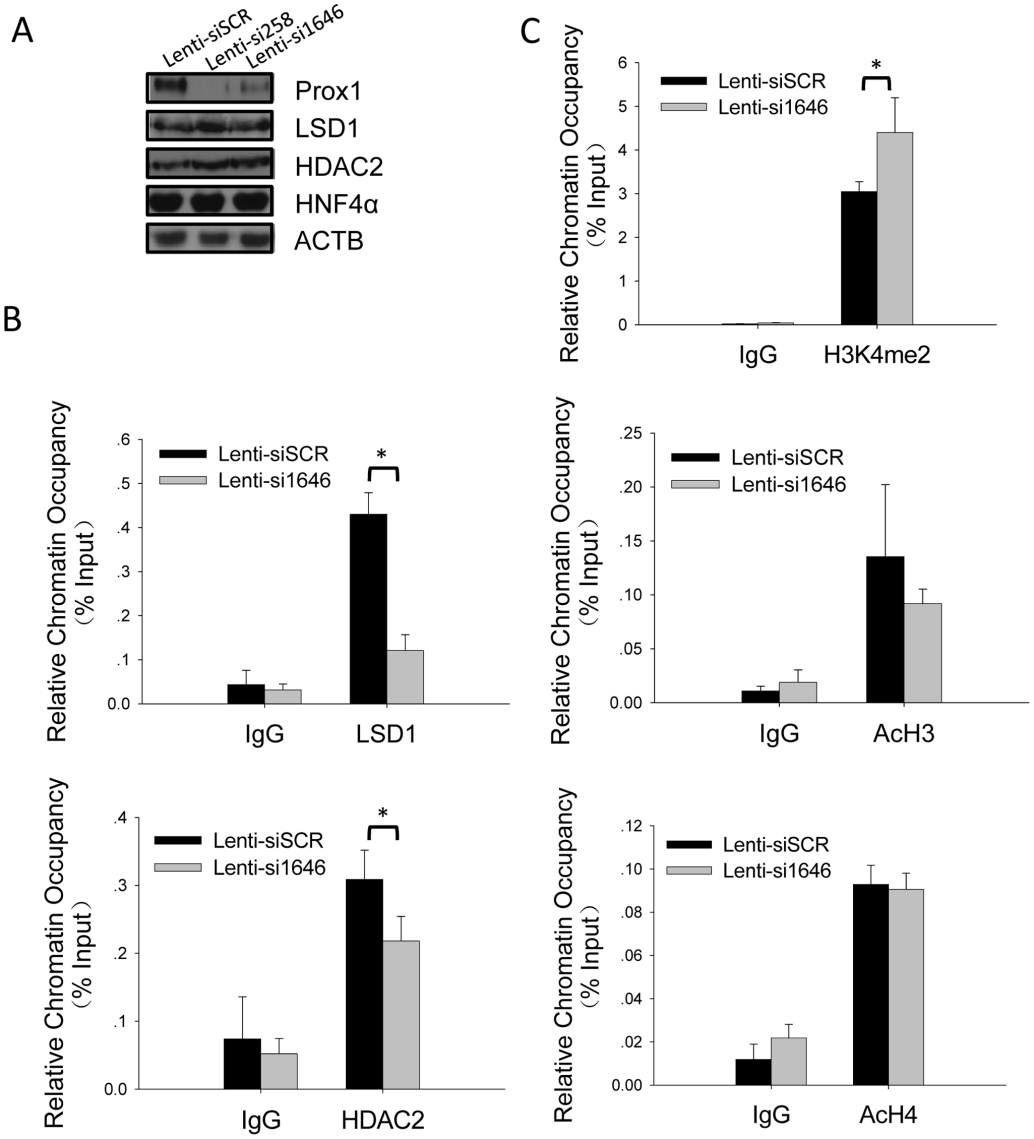
Prox1 recruits LSD1/NuRD complex components to CYP7A1 promoter and engenders repressive epigenetic changes in histone modification patterns. (A) Expression levels of LSD1/NuRD complex components LSD1 and HDAC2 in HepG2 are not affected by Prox1. HepG2 cells were infected with recombinant lentiviruses expressing Prox1-targeting siRNA precursors si258 or si1646, or scrambled control siSCR as indicated and protein levels of Prox1, HNF4α, LSD1 and HDAC2 were detected in Western blot 36 hours post infection. Beta-actin was used as loading control. (B) Knockdown of Prox1 decreases LSD1 and HDAC2 occupancy on *CYP7A1* promoter. HepG2 cells infected with indicated recombinant lentiviruses were subjected to ChIP analysis using antibodies to LSD1 and HDAC2, respectively. (C) Knockdown of Prox1 increases the level of H3K4 methylation on *CYP7A1* promoter. HepG2 cells infected with indicated recombinant lentiviruses were subjected to ChIP analysis using antibodies to di-methylated H3K4 (H3K4me2), acetylated H3 and acetylated H4, respectively. Precipitated *CYP7A1* promoter segments in B and C were detected using quantitative real-time PCR and relative chromatin occupancy was calculated as %input as described in Materials and Methods. Normal mouse/rabbit IgG was used as non-specific control. Means and SD from three independent experiments are presented. Statistically significant changes (*P<*0.05 in student’s *t* test) were indicated (*). Results similar to B and C were obtained using lenti-si258 infection (Supplementary Figure S1).

Association of Prox1 and LSD1/NuRD complex in hepatocytes and their co-localization on *CYP7A1* promoter suggest that Prox1 could recruit the repressive complex for co-repressing *CYP7A1* transcription. To provide further evidence for such recruitment, we went on to investigate whether knockdown of endogenous Prox1 expression would reduce LSD1/NuRD complex occupancy on *CYP7A1* promoter. Infection of HepG2 cells with recombinant lentiviruses expressing Prox1-targeting siRNA precursors lenti-si258 or lenti-si1646 effectively knocked down endogenous Prox1 expression, without markedly affecting LSD1 or HDAC2 expression (Fig. 4A). At the same time, a significant reduction in LSD1 occupancy on *CYP7A1* promoter was observed (Fig. 4B, top). Occupancy of HDAC2 on *CYP7A1* promoter also decreased, although less markedly (Fig. 4B, bottom). These results demonstrated that Prox1 does indeed recruit LSD1/NuRD complex components onto *CYP7A1* promoter and also suggested that HDAC2 might be additionally recruited through other mechanisms.

Functional significance of Prox1-mediated recruitment of LSD1/NuRD complex was then analyzed by examining changes in LSD1/NuRD-catalyzed nucleosomal histone modifications at *CYP7A1* promoter as a result of Prox1 knockdown. LSD1 catalyzes the demethylation of H3K4me2 and in HepG2 with endogenous Prox1 knocked down, decrease of LSD1 occupancy on *CYP7A1* promoter (Fig. 4B, top) was accompanied by a significant increase of H3K4me2 presence at the same region (Fig. 4C, top). Increased H3K4 methylation level is associated with higher transcriptional activity [37] and as has already been shown, knockdown of Prox1 indeed resulted in elevated *CYP7A1* transcription (Fig. 1). HDAC2-catalyzed deacetylation of acetylated H3 (AcH3) and H4 (AcH4) on *CYP7A1* promoter, on the other hand, was not markedly affected by Prox1 knockdown (Fig. 4C, middle and bottom). This is probably a reflection of the moderate decrease of HDAC2 occupancy in response to decreased Prox1 expression (Fig. 4B, bottom).

Taken together, these data demonstrated that Prox1 recruits the repressive LSD1/NuRD complex to *CYP7A1* promoter and demethylation of H3K4me2 by LSD1, possibly in combination with other enzymatic activities possessed by the complex, contributes towards epigenetically repressing transcription initiated from *CYP7A1* promoter. Such epigenetic mechanisms provide novel insights into Prox1-mediated co-repression.

### Involvement of Prox1-mediated LSD1/NuRD Complex Recruitment in Transcriptional Repression of *CYP7A1* in Response to Bile Acids

**Figure 5:**
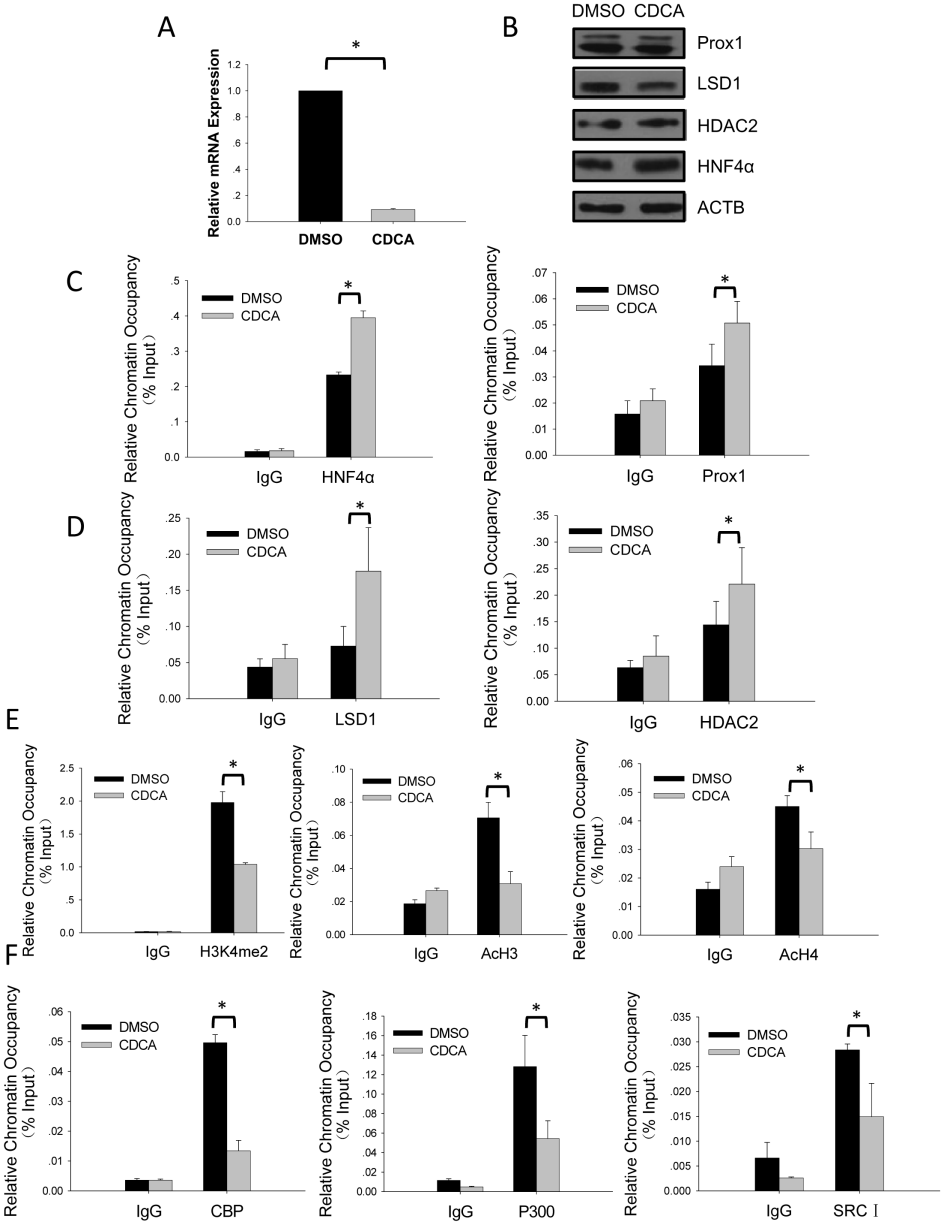
Prox1-mediated recruitment of LSD1/NuRD complex to *CYP7A1* promoter participates in bile acids induced repression of *CYP7A1*. (A) Chenodeoxycholic acid (CDCA) treatment of HepG2 cells results in repression of *CYP7A1* transcription. Total RNA from HepG2 cells treated with 25 µmol/L CDCA or DMSO vehicle for 16 hours was subjected to quantitative real-time PCR analysis of *CYP7A1* mRNA. (B) Expression levels of Prox1, LSD1 and HDAC2 in CDCA-treated HepG2. HepG2 cells treated with CDCA or DMSO vehicle were subjected to Western blot analysis using indicated antibodies. Beta-actin was used as loading control. (C) CDCA-treatment increases HNF4α and Prox1 occupancy on *CYP7A1* promoter. (D) CDCA-treatment increases LSD1 and HDAC2 occupancy on *CYP7A1* promoter. (E) CDCA-treatment decreases the level of H3K4 methylation and H3/H4 acetylation on *CYP7A1* promoter. (F) Detachment of co-activators from *CYP7A1* promoter in response to CDCA treatment. In panels C-F, HepG2 cells treated with CDCA or DMSO vehicle were subjected to ChIP analysis using indicated antibodies. Precipitated *CYP7A1* promoter segments were detected using quantitative real-time PCR and relative chromatin occupancy was calculated as %input as described in Materials and Methods. Normal mouse/rabbit IgG was used as non-specific control. Means and SD from three or six (ChIP using Prox1 and HDAC2 antibodies) independent experiments are presented. Statistically significant changes (*P<*0.05 in student’s *t* test) were indicated (*).

Negative feedback regulation of *CYP7A1* transcription in hepatocytes imposed by BA involves multiple pathways and mechanisms, many of which eventually target the two main transcription activators FTF and HNF4α [1], [2]. As previous reports have shown that Prox1 co-represses both FTF and HNF4α [27], [28], it is possible that Prox1-mediated epigenetic co-repression through LSD1/NuRD complex recruitment might be involved in BA-induced *CYP7A1* repression. To test this hypothesis, HepG2 cells were treated with CDCA and a significant decrease of *CYP7A1* mRNA level was observed (Fig. 5A), in agreement with previous results [33]. Expression levels of HNF4α, Prox1 and HDAC2 displayed no marked changes in CDCA-treated cells, whereas LSD1 expression slightly decreased (Fig. 5B). When ChIP was used to analyze these factors’ occupancy on *CYP7A1* promoter, however, both HNF4α and Prox1 displayed significantly increased occupancy in response to CDCA treatment (Fig. 5C). LSD1 and, to a lesser extent, HDAC2 occupancy also increased, (Fig. 5D), most likely a result of elevated recruitment of LSD1/NuRD complex by Prox1. Increased occupancy of LSD1/NuRD complex on *CYP7A1* promoter in turn resulted in decreased H3K4 methylation and H3/H4 acetylation levels at the promoter region (Fig. 5E). Such changes in histone modification status represented a transition of local chromatin configuration from a more transcriptionally active state towards a more transcriptionally repressive state, which is also reflected in significant detachment of co-activators including CBP, p300 and SRC-1 from *CYP7A1* promoter (Fig. 5F). These data confirmed that Prox1-mediated co-repression of *CYP7A1* promoter through LSD1/NuRD complex recruitment could indeed participate in BA-induced repression of *CYP7A1* transcription.

## Discussion

Prox1 is a co-repressor for both of the two key factors responsible for regulating *CYP7*A1 transcription, namely FTF [27] and HNF4α [28], and functionally represses CYP7A1 expression and bile acid synthesis in hepatocytes (Fig. 1). In this work, mechanisms involved in Prox1-mediated co-repression of *CYP7*A1 transcription were explored using IP-MS methodology (Fig. 2A). Prox1 was demonstrated to associate with multiple components of LSD1/NuRD complex (Fig. 2B and 2C), most likely through interacting directly with LSD1 (Fig. 2D). ChIP and sequential ChIP assays showed that Prox1 co-localizes with LSD1/NuRD complex components on *CYP7A1* promoter (Fig. 3), and such co-localization is the result of Prox1-mediated recruitment (Fig. 4A and 4B). Recruitment of LSD1/NuRD complex by Prox1 engenders repressive changes in chromatin histone modifications at *CYP7A1* promoter (Fig. 4C) that contribute towards repression of transcription. Finally, Prox1-mediated LSD1/NuRD complex recruitment is involved in negative feedback repression of *CYP7A1* transcription by bile acids (Fig. 5). These data revealed novel epigenetic mechanisms employed by Prox1 to co-repress *CYP7A1* promoter and reiterated the significance of epigenetic regulation in modulating *CYP7A1* transcription.

Association with directly DNA-binding transcription factors followed by recruitment of other functional factors or bridging adaptors is a common mechanism through which many co-repressors and co-activators exert their effects on target promoters. Although Prox1 has been shown to co-regulate expression of multiple genes, including *CYP7A1*, through interacting with DNA-binding factors like FTF [27] and HNF4α [28], little has been known regarding what and how other factors are involved. Direct interaction of Prox1 with LSD1 (Fig. 2D) apparently enables Prox1 to recruit the repressive chromatin-modifying LSD1/NuRD complex (Fig. 3 and 4B), which in turn engenders histone modification changes at target gene promoter indicative of epigenetic silencing (Fig. 4C). Preliminary delineation of Prox1-LSD1 interactions indicated that both the N-terminal repression domain and the C-terminal homeobox/Prospero domain of Prox1 are capable of binding LSD1 (Fig. 2D). Previous results have shown that the repression domain is also responsible for binding FTF [27] and HNF4α [28]. It is therefore possible that Prox1 utilizes N-terminal repression domain for binding to DNA-bound transcription factors, while recruiting LSD1 and other factors through its C-terminal homeobox/Prospero domain.

LSD1/NuRD is a repressive complex abundantly present in most cell and tissue types. LSD1/NuRD couples histone deacetylase (HDAC1/HDAC2), histone demethylase (LSD1) and chromatin remodeling ATPase (Mi-2 α and β) activities in a single complex. In addition, the complex also possesses methylated DNA-binding activities through the MBD2/3 components [34], [38]. The combined activities of the enzymatic components of LSD1/NuRD complex are capable of converting an active, hyperacetylated and H3K4-hypermethylated promoter region into densely packed, hypoacetylated and H3K4-hypomethylated nucleosomes, characteristic of transcriptionally inactive chromatin. Recruitment of such a potent epigenetic regulator complex clearly enables Prox1 to achieve marked co-repression of *CYP7A1* in hepatocytes (Fig. 4). Considering the wide distribution of LSD1/NuRD complex among cell and tissue types, it is likely that Prox1 might recruit LSD1/NuRD complex to regulate other target genes as well, in both hepatocytes and non-hepatocytes. Further research is warranted to address such possibilities.

Multiple epigenetic mechanisms have been shown to be involved in the regulation of *CYP7A1* transcription. For instance, SHP is another key co-repressor of *CYP7A1*, and like Prox1, SHP interacts with both FTF and HNF4α to repress their trans-activation of *CYP7A1* promoter [14], [15]. SHP was found to recruit the mSin3A-Swi/Snf complex, which possesses both HDAC and chromatin remodeling ATPase activities, to *CYP7A1* promoter and render transcriptional inhibition [33]. In addition, SHP was also reported to interact functionally with HDAC1 and the euchromatic H3K9 methlyltransferase G9a, which might enables SHP to silence transcriptionally active promoters [39]. In BA-induced repression of *CYP7A1*, recruitment of a series of epigenetic regulators including HDAC7, HDAC3, HDAC1, SMRTα and NCoR to *CYP7A1* promoter could be observed following BA treatment, and the recruited HDAC activities were shown to be essential for transcriptional silencing of *CYP7A1* [40]. Results from this work provide evidences for the participation, through Prox1, of more epigenetic factors and mechanisms in the regulation of *CYP7A1* transcription in hepatocytes. It is obvious that there exist functional overlaps and probably functional redundancies among these different epigenetic pathways.

Despite the lack of appreciable changes in HNF4α expression levels in BA treated HepG2 cells (Fig. 5B), occupancy of HNF4α on *CYP7A1* promoter increased significantly (Fig. 5C). How this could have been achieved in the cells is intriguing. One possibility is that BA-dependent signaling somehow removed factors interfering with HNF4α’s binding from *CYP7A1* promoter. Since HNF4α and FTF bind to overlapping sites on *CYP7A1* promoter [41], whether FTF could be involved in such BA-induced transcription factor reconfiguration at *CYP7A1* promoter region warrants further investigation. An alternative but not mutually exclusive explanation could be that BA treatment somehow enhanced HNF4α’s affinity for its cognitive binding site. Whatever the underlying mechanisms, increased HNF4α binding apparently recruited more Prox1 co-repressor to *CYP7A1* promoter (Fig. 5C), even though Prox1 expression level was also unchanged by BA treatment (Fig. 5B). Prox1 in turn recruited more LSD1/NuRD complex components including LSD1 and HDAC2 (Fig. 5D), which engendered repressive epigenetic modifications to chromatin histones (Fig. 5E). Meanwhile, detachment of transcription co-activators from *CYP7A1* promoter could be observed (Fig. 5F), consistent with decreased *CYP7A1* transcription (Fig. 5A). It should be noted that although Prox1-mediated LSD1/NuRD complex recruitment clearly participates in such a process, especially for LSD1-catalyzed H3K4 de-methylation, other factors and mechanisms with similar histone de-acetylation and chromatin remodeling functions are no doubt also at work (see previous paragraph), although not necessarily on the same single *CYP7A1* promoter at the same time. These results highlighted the complexities involved in the modulation of one of the most important enzymes in BA metabolism.

## Supporting Information

Figure S1 **Knockdown of Prox1 decreases LSD1 and HDAC2 occupancy on** ***CYP7A1*** **promoter and increases the level of H3K4 methylation on** ***CYP7A1*** **promoter.** HepG2 cells infected with recombinant lentiviruses expressing Prox1-targeting siRNA precursors si258, or scrambled control siSCR as indicated, were subjected to ChIP analysis using antibodies to LSD1, HDAC2 and di-methylated H3K4 (H3K4me2) respectively. Precipitated *CYP7A1* promoter segments were detected using quantitative real-time PCR and relative chromatin occupancy was calculated as %input as described in Materials and Methods. Normal mouse/rabbit IgG was used as non-specific control. Means and SD from three independent experiments are presented. Statistically significant changes (*P<*0.05 in student’s *t* test) were indicated (*).
(PDF)

Figure S2 **Association of endogenous Prox1 with LSD1/NuRD complex in HepG2 cells.** HepG2 cells were subjected to co-immunoprecipitation assay using anti-Prox1 antibodies in the presence of DNaseI (0.1 µg/µl) and RNaseA (0.2 µg/µl). Co-immunoprecipitated HNF4α and LSD1/NuRD complex components were detected in Western blot using corresponding antibodies as indicated.
(PDF)

Table S1 **LSD1/NuRD complex components identified by mass spectrometry in proteins co-immunoprecipitated with Prox1.**
(PDF)
