# Supplementary material for: Prox1 Directly Interacts with LSD1 and Recruits the LSD1/NuRD Complex to Epigenetically Co-Repress CYP7A1 Transcription
Source: PLoS One. 2013 Apr 23;8(4):e62192. doi: 10.1371/journal.pone.0062192 (PMC3633876; doi:10.1371/journal.pone.0062192)
Supplement: Figure S1 — Knockdown of Prox1 decreases LSD1 and HDAC2 occupancy on CYP7A1 promoter and increases the level of H3K4 methylation on CYP7A1 promoter. HepG2 cells infected with recombinant lentiviruses expressing Prox1-targeting siRNA precursors si258, or scrambled control siSCR as indicated, were subjected to ChIP analysis using antibodies to LSD1, HDAC2 and di-methylated H3K4 (H3K4me2) respectively. Precipitated CYP7A1 promoter segments were detected using quantitative real-time PCR and relative chromatin occupancy was calculated as %input as described in Materials and Methods. Normal mouse/rabbit IgG was used as non-specific control. Means and SD from three independent experiments are presented. Statistically significant changes (P<0.05 in student’s t test) were indicated (*). (PDF) [file pone.0062192.s001.pdf]

Supplementary Figure

S1

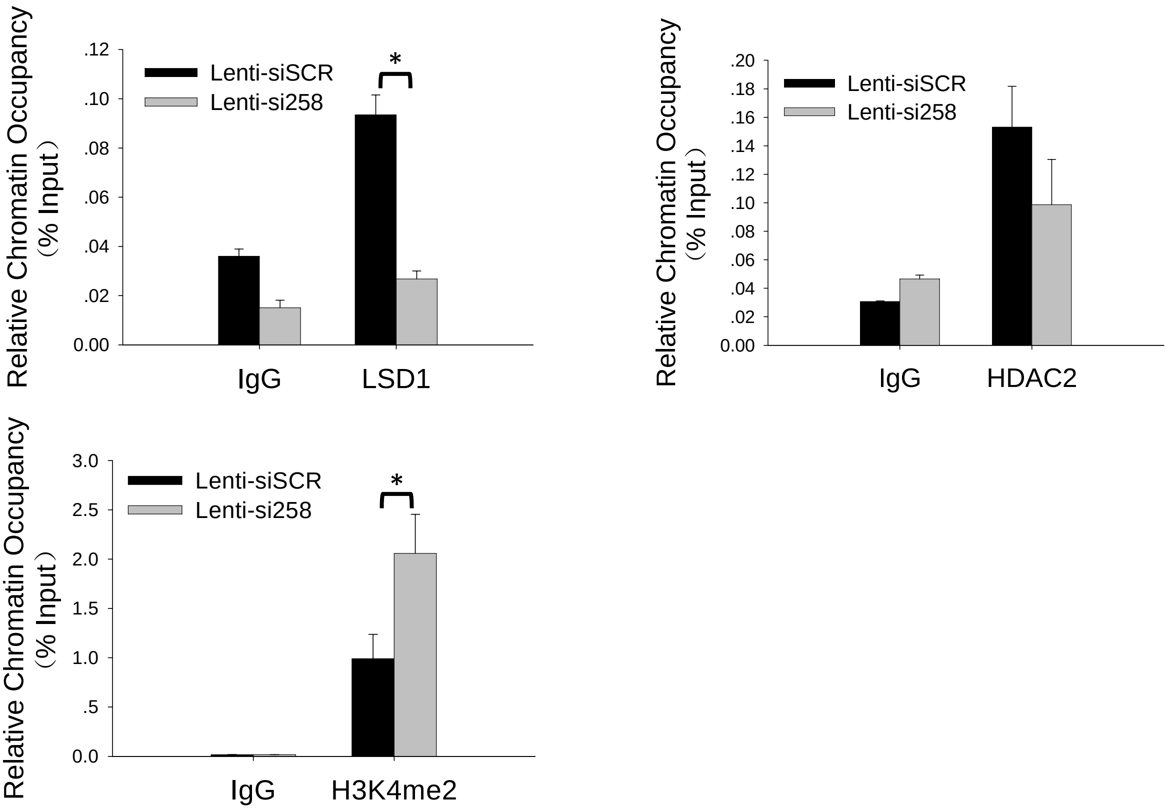

Figure S1. Knockdown of Prox1 decreases LSD1 and HDAC2 occupancy on *CYP7A1* promoter and increases the level of H3K4 methylation on *CYP7A1* promoter. HepG2 cells infected with recombinant lentiviruses expressing Prox1-targeting siRNA precursors si258, or scrambled control siSCR as indicated, were subjected to ChIP analysis using antibodies to LSD1, HDAC2 and di-methylated H3K4 (H3K4me2) respectively. Precipitated *CYP7A1* promoter segments were detected using quantitative real-time PCR and relative chromatin occupancy was calculated as %input as described in Materials and Methods. Normal mouse/rabbit IgG was used as non-specific control. Means and SD from three independent experiments are presented. Statistically significant changes ($P < 0.05$ in student's t test) were indicated (*).
