# Supplementary material for: Prox1 Directly Interacts with LSD1 and Recruits the LSD1/NuRD Complex to Epigenetically Co-Repress CYP7A1 Transcription
Source: PLoS One. 2013 Apr 23;8(4):e62192. doi: 10.1371/journal.pone.0062192 (PMC3633876; doi:10.1371/journal.pone.0062192)
Supplement: Figure S2 — Association of endogenous Prox1 with LSD1/NuRD complex in HepG2 cells. HepG2 cells were subjected to co-immunoprecipitation assay using anti-Prox1 antibodies in the presence of DNaseI (0.1 µg/µl) and RNaseA (0.2 µg/µl). Co-immunoprecipitated HNF4α and LSD1/NuRD complex components were detected in Western blot using corresponding antibodies as indicated. (PDF) [file pone.0062192.s002.pdf]

Supplementary Figure

S2

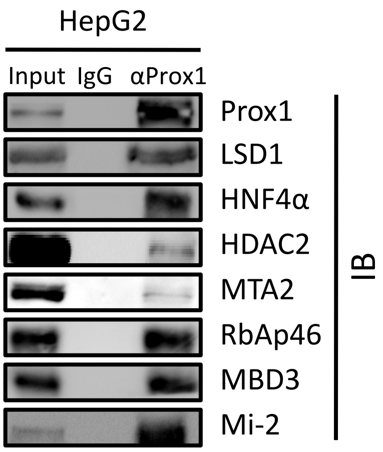

Figure S2. Association of endogenous Prox1 with LSD1/NuRD complex in HepG2 cells. HepG2 cells were subjected to co-immunoprecipitation assay using anti-Prox1 antibodies in the presence of DNaseI (0.1μg/μl) and RNaseA (0.2μg/μl). Co-immunoprecipitated HNF4a and LSD1/NuRD complex components were detected in Western blot using corresponding antibodies as indicated.
