# Supplementary material for: Prox1 Directly Interacts with LSD1 and Recruits the LSD1/NuRD Complex to Epigenetically Co-Repress CYP7A1 Transcription
Source: PLoS One. 2013 Apr 23;8(4):e62192. doi: 10.1371/journal.pone.0062192 (PMC3633876; doi:10.1371/journal.pone.0062192)
Supplement: Table S1 — LSD1/NuRD complex components identified by mass spectrometry in proteins co-immunoprecipitated with Prox1. (PDF) [file pone.0062192.s003.pdf]

Supporting Information:

Table S1. LSD1/NuRD complex components identified by mass spectrometry in proteins co-immunoprecipitated with Prox1.

Peptides derived from LSD1/NuRD complex components MTA2, HDAC2, RbAp46 and MBD3 are listed. Prox1-derived peptides are also shown as indicator of successful IP. Data are compiled from raw data generated by ABI 4700 MALDI TOF/TOF.

Protein Name	Accession No.	Protein MW	Protein PI	Pep. Count	Protein score	
**Prospero homeobox protein 1(Prox1)**	**gi/85702224/sp/Q92786.2/PROX1_H**	**83151**	**6.74**	**10**	**50**	
Peptide Information						
Calc.Mass	Obsrv.Mass	±da	±ppm	Start Seq.	End Seq.	Sequence
856.41	856.3257	-0.0843	-98	623	628	WFSNFR
859.4196	859.3539	-0.0657	-76	605	611	TYFSDVK
993.4862	993.3897	-0.0965	-97	590	596	LMFFYTR
1076.5007	1076.3979	-0.1028	-95	672	680	ANDFEVPER
1652.7697	1652.7572	-0.0125	-8	192	205	EMAPQSVSPRESYR
1717.9747	1717.8182	-0.1565	-91	378	392	QVPQVFPPLQIPQAR
1833.9188	1833.7462	-0.1726	-94	641	657	QAINDGVTSTEELSITR
1855.8583	1855.6876	-0.1707	-92	393	408	FAVNGENHNFHTANQR
1856.8667	1856.6816	-0.1851	-100	175	191	GMSHSPSVALRGNENER
1884.7738	1884.5905	-0.1833	-97	295	310	SDNEMCELDPGQFIDR
**Protein Name**	**Accession No.**	**Protein MW**	**Protein PI**	**Pep. Count**	**Protein score**	
**Metastasis-associated protein MTA2**	**gi/29840793/sp/O94776/MTA2_HUMA**	**74975.7**	**9.7**	**10**	**58**	
Peptide Information						
Calc.Mass	Obsrv.Mass	±da	±ppm	Start Seq.	End Seq.	Sequence
1073.5586	1073.4552	-0.1034	-96	406	415	TPTQLEGATR
1115.6055	1115.4988	-0.1067	-96	139	148	TLLADQGEIR
1188.5977	1188.5594	-0.0383	-32	468	476	MCRDLLQPR
1298.6852	1298.5753	-0.1099	-85	93	103	QFESLPATHIR
1327.6277	1327.6053	-0.0224	-17	178	188	VWDPDNPLTDR

1549.7452	1549.603	-0.1422	-92	50	64	DISSSLNSLADSNAR
1576.7788	1576.6472	-0.1316	-83	228	240	DITLFHAMDTLQR
1906.8777	1906.7061	-0.171	-90	65	80	EFEEESKQPGVSEQQR
2108.9922	2108.7957	-0.1965	-93	8	25	VRGDYVYFENSSSNPYLVR
2172.0791	2171.8799	-0.1992	-92	424	443	GNRHLSRPEAQSLSPYTTSANR
**Protein Name**	**Accession No.**	**Protein MW**	**Protein PI**	**Pep. Count**	**Protein score**	
**Histone deacetylase 2 (HD2)**	**gi/68068066/sp/Q92769/ HDAC2_HUM**	**55328.8**	**5.59**	**6**	**70**	
Peptide Information						
Calc.Mass	Obsrv.Mass	±da	±ppm	Start Seq.	End Seq.	Sequence
1073.556	1073.5632	0.0072	7	52	59	MEIYRPHK
1374.6324	1374.7098	0.0774	56	202	213	YGEYFPGTGDLR
1426.6631	1426.7743	0.1112	78	79	90	SIRPDNMSEYSK
1607.8363	1607.9253	0.089	55	38	50	MTHNLLLNYGLYR
1915.9185	1916.0317	0.1132	59	202	219	YGEYFPGTGDLRDIGAGK
2678.2732	2678.5112	0.238	89	171	193	VLYIDIDIHHGDGVEEAFYTTDR
**Protein Name**	**Accession No.**	**Protein MW**	**Protein PI**	**Pep. Count**	**Protein score**	
**Histone binding protein RBBP7(RbAp46)**	**gi/249489/sp/Q1657 6.1/RBBP7_HU**	**47790.2**	**4.89**	**9**	**65**	
Peptide Information						
Calc.Mass	Obsrv.Mass	±da	±ppm	Start Seq.	End Seq.	Sequence
950.4764	950.4092	-0.0672	-71	251	257	LMIWDTR
973.5465	973.4631	-0.0834	-86	296	303	TVALWDLR
1067.5228	1067.4398	-0.083	-78	120	128	INHEGEVNR
1130.6317	1130.5287	-0.103	-91	340	348	RLNVWDLSK
1284.5413	1284.4216	-0.1197	-93	5	14	EMFEDTVEER
1428.7303	1428.598	-0.1322	-93	131	142	YMPQNPHIIATK
1471.7314	1471.5929	-0.1385	-94	143	155	TPSSDVLVFDYTK
1701.7458	1701.7502	0.0044	3	1	14	MASKEMFEDTVEER
2847.343	2847.127	-0.216	-76	349	375	IGEEQSAEDAEDGPPELLFIHGGHTAK
**Protein Name**	**Accession No.**	**Protein MW**	**Protein PI**	**Pep. Count**	**Protein score**	
**Methyl-CpG-binding domain protein 3(MBD3)**	**gi/50400820/sp/ O95983/MBD3_ HUMA**	**32823.3**	**5.22**	**6**	**39**	
Peptide Information						
Calc.Mass	Obsrv.Mass	±da	±ppm	Start Seq.	End Seq.	Sequence
978.5406	978.5549	0.0143	15	136	142	QLFWEKK

1256.6957	1256.6705	-0.0252	-20	227	236	KQEELVQQVR
1256.6957	1256.6705	-0.0252	-20	228	237	QEELVQQVRK
1516.7906	1516.8689	0.0783	52	130	141	AVDQPRQLFWEK
2081.9861	2082.137	0.1509	72	6	22	WECPALPQGWEREEVPR
2197.1104	2197.1646	0.0542	25	238	256	RLEEALMADMLAHVEELAR
